# The prevalence of suicide ideation and predictive factors among pregnant women in the third trimester

**DOI:** 10.1186/s12884-022-04590-6

**Published:** 2022-03-29

**Authors:** Ling Zhang, Yating Yang, Mengdie Li, Xiaoqin Zhou, Kai Zhang, Xuai Yin, Huanzhong Liu

**Affiliations:** 1 Department of Psychiatry, Chaohu Hospital of Anhui Medical University, Hefei, 238000 China; 2 Department of Obstetric and Gynecology, Chaohu Hospital of Anhui Medical University, Hefei, 238000 China

**Keywords:** Suicide ideation, Predictive factors, The third trimester, Attachment

## Abstract

**Background:**

Pregnancy is a period for women undergo major physical and psychological changes. Suicide is a cause of maternal death and suicidal ideation is a key factor in suicidal behavior. The purpose of this study was to investigate the prevalence of suicidal ideation in the third trimester and associated predictors including psychological factors such as attachment.

**Methods:**

A cross-sectional study included 432 pregnant women in the third trimester of pregnancy was conducted in a tertiary hospital. The Edinburgh Postpartum Depression Scale (EPDS) was used to assess prenatal depression and suicidal ideation. The Zung Self-Rating Anxiety Scale (SAS) and Experience of Close Relationship (ECR) scale were used to assess anxiety and attachment respectively.

**Results:**

The results showed that the EPDS scale screened 6.71% of pregnant women with suicidal ideation. Compared with those without suicidal ideation, pregnant women with suicidal ideation had a higher prevalence of insecure attachment, higher scores on the two dimensions of attachment (attachment avoidance and attachment anxiety), and higher prevalence of prenatal depression and anxiety. Binary logistic regression showed that marital satisfaction was a protective factor for suicidal ideation, while prenatal depression, prenatal anxiety and attachment anxiety were risk factors for suicidal ideation.

**Conclusions:**

The suicidal ideation among pregnant women was high, which should be given more attention. In the process of preventing and intervening suicidal ideation, in addition to the emotional state of pregnant women, their psychological factors such as attachment anxiety should also be considered.

## Introduction

Suicide is a devastating public health problem for individuals, families and society [1, 2]. Before reaching the final suicidal behavior, people may experience from suicidal ideation to suicide attempts [3]. Suicidal ideation is considered to be a key predictive factor of subsequent suicide attempts and suicide completion [4]. Pregnancy is a major stressful life event for women and suicide is one of the main causes of maternal mortality [5]. According to a systematic review, suicidal ideation was a common complication of pregnancy and pregnant women were more likely to have suicidal ideation than the general population [6]. Prenatal suicidal ideation in pregnant women may increase postpartum depression and suicidal ideation rate [7]. Moreover, prenatal suicidal ideation was closely related to adverse birth outcomes such as low birth weight and lower neurodevelopmental score in infants [8, 9]. Therefore, it is necessary to investigate the suicidal ideation of pregnant women to prevent prenatal suicidal behavior and corresponding postpartum health behaviors.

Some factors that contributed to suicidal ideation during pregnancy, such as abuse and partner violence during pregnancy, low social support [10, 11]. In addition, researchers have demonstrated that the molecular precursors of neurotransmitters serotonin and dopamine were positively correlated with suicidal ideation, while thiamine was negatively correlated with suicidal ideation [12]. Furthermore, a systematic review indicated that mental disorders during pregnancy are significant risk factors for suicidal ideation in pregnant women [6].

Due to the physical and psychological changes caused by pregnancy, it poses a huge challenge to the mental health of pregnant women, which can lead to various mental disorders, such as prenatal depression and anxiety [13, 14]. Yin et al. indicated that the combined prevalence of antenatal depression during pregnancy was 20.7% [15]. A meta-analysis of African pregnant women found that up to 26% of pregnant women suffered from prenatal depression [16]. The prevalence of antenatal anxiety has been reported to range from approximately 18% in the first trimester to 25% in the third trimester [17]. Perinatal depression and anxiety are associated with increased maternal mortality [18].

Apart from the influencing factors mentioned above, some personality traits and trait anxiety are associated with increased perinatal suicidal ideation [19]. It has been proposed that a kind of psychological factor such as attachment goes beyond personality traits in predicting psychological risk in pregnant women [20]. A study of 233 prospective parents showed that attachment security was associated with mental health including suicidal ideation and emotional distress [21]. Attachment refers to the intimate relationship between a person and the original caregiver, which produces an internal working model in the process of attachment formation. The internal working model is activated in the event of a stressful event such as pregnancy [22]. Insecure attachment may increase the impact of stressful life events on individuals, leading to the emergence of pathological psychology [23]. A study involving medical students showed that attachment style can be used as a variable to regulate suicidal thoughts [24] and insecure attachment linked to increased risk of suicide [25].

The third trimester is the most physically burdensome period for pregnant women, and a previous study has found that pain in the third trimester affects more than two-thirds of pregnant women [26]. A 15-year perinatal study of pregnant women in Canada found that suicide rate was highest in the third trimester compared to other trimesters [27]. Moreover, pregnant women in the third trimester are faced with the fear of childbirth. It is very important to pay attention to the psychological state and suicide risk during this period.

The purpose of this article was to investigate the prevalence of suicidal ideation and predictive factors including attachment in pregnant women in the third trimester of pregnancy. We hypothesized that maternal mood and attachment could predict maternal suicidal ideation in the third trimester.

## Methods

### Participants and procedure

This was a cross-sectional study conducted in Chaohu Hospital of Anhui Medical University from September 2019 to November 2019. The hospital is a tertiary hospital affiliated to the university and provides medical care for about one million people. Our participants were pregnant women from the obstetrical clinic of the hospital who met the following criteria: 1)18–40 years old; 2) gestational age of 28–40 weeks; 3) singleton pregnancy. The exclusion criteria for participants were as below: 1) Previous history of mental disorders; 2) With complications during pregnancy (gestational diabetes, hypertension, and pre-eclampsia; 3) Previous history of miscarriage.

We used a single sample to estimate the sample size. *N* = Z^2^ × (P × (1-P))/E^2^. When α = 0.05, the confidence interval is 95%, Z = 1.96, the allowable error of the sample E = 5%, and *P* = 0.5, so that N = 384. Considering the 10% non-response rate, the minimum sample that should be accepted is 423. In the end we included a total of 432 pregnant women.

All researchers conducted systematic standardized training on the project before the start of the research. A brief introduction to the study was given to all pregnant women in their third trimester who attended the obstetric clinic. Finally, according to the inclusion and exclusion criteria of the study, a total of 432 pregnant women who met the criteria were included. There would be enough time to make a decision before they decide whether to participate in the study. All participants completed the scales on their own after being instructed by uniformly trained investigators. All pregnant women provided written informed consent. The ethics committee of Chaohu Hospital of Anhui Medical University authorized the study.

### Instruments

#### Demographic data

A standard demographic questionnaire was used by uniformly trained researchers to collect demographic data of pregnant women, such as age, gestational week, education, parity, working status, planned pregnancy and marital satisfaction. With the exception of age, gestational age and education, the following standardized questions asked about other demographically relevant factors: “Are you pregnant for the first time?”; “Is the pregnancy planned?”; “Did you keep working during the pregnancy?”; “Are you satisfied with your marriage?” All questions will be answered by selecting "Yes" or "No".

#### Prenatal depression and suicidal ideation

The Edinburgh Postpartum Depression Scale (EPDS) [28] was used to evaluate maternal depression symptoms. There are ten items in the scale, each of which has a score of 0 (No, not at all) to 3 (Yes, all the time), with a full score of 30. A higher total score means more severe depressive symptoms. The cutoff value of EPDS in this study was 12, which has been validated and applied in Chinese culture [29–31]. Suicidal ideation was measured by the EPDS item 10, “I have thought of hurting myself”. Item 10 of the EPDS reflects language commonly used to assess suicidal ideation in clinical interviews [32]. A score of 0 for this item is considered as having no suicidal ideation, other answers are considered as having suicidal ideation. These binary variables for suicidal ideation have been applied to studies in different populations with the EPDS item 10 [33–35].

#### Attachment

The Experience of Close Relationship (ECR) [36] was used to evaluate attachment of pregnant women. The scale has been confirmed to be reliable and used in Chinese people [37]. There are 36 items, including attachment anxiety and attachment avoidance, each with 18 items. According to the scores of attachment anxiety, it can be divided into four tyles: secure (low anxiety and low avoidance attentive), attentive (low anxiety and high avoidance), indifferent (high avoidance and low anxiety) and phobic (high avoidance and high anxiety), of which the latter three are classified as insecure attachment [38].

#### Prenatal anxiety

The Zung Self-Rating Anxiety Scale (SAS) [39] was used to assess the prenatal anxiety during pregnancy. There are 20 items in the scale, and each item is scored in 4 levels. The total score multiplied by 1.25 is the final standard score. The cut-off for this study is the standard score ≥ 50, which has been validated and applied in previous Chinese studies [40, 41]. The lower the total score, the fewer anxiety symptoms.

### Data analysis

All data were analyzed with the Statistical Package for Social Sciences 23.0 (SPSS23.0). We compared the differences in basic demographics, attachment and mental health variables between pregnant women with or without suicidal ideation. The t-test or Mann–Whitney U test was used to compare continuous variables according to the normality of continuous variables and chi-square test was used to compare categorical variables. After controlling for confounding factors, binary regression analysis (univariable and multivariable)was used to analyze important predictive factors related to suicidal ideation. In multivariate logistic regression model, suicidal ideation was the dependent variable, the covariates were the variables that showed significant differences between the suicidal ideation group and no suicidal ideation group. *P* < 0.05 is statistically significant.

## Results

### Demographic data of pregnant women

Altogether, 432 pregnant women who met the study criteria were enrolled in this study. All participants were in the third trimester, with an average gestational age of 35.57(SD = 2.60) weeks, and an average age of 28.49(SD = 4.37)years old. The education level of most participants (51.39%) was high school or junior college. In addition, 60.19%% of participants were nulliparous women. The majority of participants had a planned pregnancy (62.04%) and 40.97% of them were employed. The majority of the participants were satisfied with marriage (82.18%). In addition, the prevalence of pregnant women suffering from the symptoms of prenatal depression and antenatal anxiety were 17.59% and 14.81%, respectively. We found that more than half of the(53.94%) had secure attachment style and the prevalence of suicidal ideation among all participants was 6.71%.

### Comparison of demographics, attachment, and mental health variables between pregnant women with suicidal ideation and pregnant women without suicidal ideation

As shown in Table 1, gestational weeks in pregnant women with suicidal ideation was higher than that without suicidal ideation (36.55 ± 3.05 vs 35.50 ± 2.55, *P* < 0.01), while the prevalence of marital satisfaction was lower than that without suicidal ideation (51.72% vs 84.37%, *P* < 0.01). The prevalence of symptoms of prenatal depression and anxiety was higher in the group with suicidal ideation than that in the group without suicidal ideation (72.4% vs 13.65%; 55.56% vs 12.16%, all *P* < 0.01). In addition, the prevalence of secure style in pregnant women with suicidal ideation was lower (31.03% vs 55.58%, *P* = 0.01) than that in the group without suicidal ideation. Besides that, both of the attachment avoidance and attachment anxiety scores were higher in the group with suicidal ideation (3.18 ± 0.65 vs 2.84 ± 0.80, *P* = 0.025; 3.58 ± 0.76 vs 2.88 ± 0.77, *P* < 0.01).

**Table 1:** Comparison of demographics, attachment, and mental health variables between pregnant women with suicidal ideation and pregnant women without suicidal ideation

	All participants (*n* = 432)	Without suicidal ideation (*n* = 403;93.29%)	With suicidal ideation (*n* = 29;6.71%)	t/Z/X^2^	P
Age (years)	28.49 ± 4.37	28.58 ± 4.45	27.17 ± 2.94	-1.364	0.173
Gestational weeks	35.57 ± 2.60	35.50 ± 2.55	36.55 ± 3.05	-2.640	< 0.01
Education level					
Less than high school	97(22.45%)	92(22.83%)	5(17.24%)	0.660	0.719
High school or junior college	222(51.39%)	207(51.36%)	15(51.73%)		
Bachelor degree or higher	113(26.16%)	104(25.81%)	9(31.03%)		
Nulliparous women	260(60.19%)	242(60.05%)	18(62.07%)	0.046	0.830
Planned pregnancy	268(62.04%)	253(62.78%)	15(51.72%)	1.404	0.236
Employed	177(40.97%)	166(41.19%)	11(37.93%)	0.119	0.730
Marital satisfaction	355(82.18%)	340(84.37%)	15(51.72%)	19.681	< 0.01
Prenatal depression	76(17.59%)	55(13.65%)	21(72.4%)	64.443	< 0.01
Prenatal anxiety	64(14.81%)	49(12.16%)	15 (55.56%)	30.496	< 0.01
Attachment style (secure)	233(53.94%)	224(55.58%)	9(31.03%)	6.562	0.010
Attachment avoidance	2.86 ± 0.79	2.84 ± 0.80	3.18 ± 0.65	2.245	0.025
Attachment anxiety	2.92 ± 0.79	2.88 ± 0.77	3.58 ± 0.76	4.764	< 0.01

### Binomial logistic regression analysis for associations with suicidal ideation

As shown in Table 2, the four statistically significant variables predicting prenatal suicidal ideation were marital satisfaction, attachment anxiety, prenatal depression and prenatal anxiety. Among them, marital satisfaction was a protective factor for suicidal ideation (*P* = 0.008, OR = 0.277, 95%CI: 0.108–0.710). And pregnant women who experienced prenatal depression (*P* < 0.001, OR = 8.231, 95%CI: 3.145–21.542), prenatal anxiety (*P* = 0.014, OR = 3.420, 95%CI: 1.280–9.137) and high attachment anxiety (*P* = 0.029, OR = 2.015, 95%CI: 1.073–3.783) were at a higher risk of suicidal ideation.

**Table 2:** Binomial logistic regression analysis for associations with suicidal ideation

	P	OR	95% CI	
			Lower	Upper
Marital satisfaction	0.008	0.277	0.108	0.710
Prenatal depression	< 0.001	8.231	3.145	21.542
Prenatal anxiety	0.014	3.420	1.280	9.137
Attachment anxiety	0.029	2.015	1.073	3.783

## Discussion

As the author knows, this was the first study to consider attachment as a predictor of suicidal ideation in pregnant women in China. We found that 6.71% of women in the third trimester had suicidal ideation, which was similar to a previous research result [42] and another study on Chinese pregnant women [30], but higher than that in Spain(2.6%) [43]. There may be some reasons for the higher suicidal ideation in our study. First of all, the participants in this study were from obstetric outpatient clinics, rather than hospitalized pregnant women, and the hospitalized environment may give pregnant women more security to welcome the arrival of new life, which in turn resulting in a lower rate of suicidal ideation. Second, our participants were all from Anhui Province of China, an economically underdeveloped region. The economy may be related to the higher prevalence of suicidal ideation. Existing research on the suicide rate of Chinese people has revealed the economic improvement of the Chinese population was related to the decline in the suicide rate [44] and financial difficulties would indirectly lead to suicidal ideation [45].

In this study, pregnant women who were satisfied with their marriage were less likely to develop suicidal ideation, which was similar to a previous study [30]. Kanako et al. also pointed out that women's mental health largely depends on their marital status [46], which emphasized the potential importance of marital satisfaction to the support network around pregnant women. A good marriage relationship is very important to promote the mental health of pregnant women and reduce suicidal ideation.

The findings revealed symptoms of prenatal depression were also a risk factor for suicidal ideation, which was consistent with findings in a study on 1517 pregnant women [35]. Previous researches also showed that suicidal ideation was associated with higher levels of depressive symptoms during prenatal and postpartum period [47, 48]. Suicidal ideation of pregnant women is usually assessed in conjunction with maternal depression. Pregnant women with more severe depressive symptoms usually have a sense of hopelessness, lose confidence in life, and thus have higher suicidal ideation. Studies have found that high rates of comorbidity between prenatal depression and suicidal ideation, more than 50% of women with prenatal suicidal ideation also reported major depressive disorder [49]. In addition, women who commit suicide in the perinatal period are more likely to suffer from untreated depression [50]. While part of participants with suicidal ideation may not suffer from depression [35], which also calls for screening for suicidal ideation in pregnant women with and without depression. In particular, the use of the depression screening scale to assess suicidal ideation in this study may miss a subset of pregnant women with suicidal ideation. This also underscores the importance of a comprehensive assessment of suicidal ideation in pregnant women in the future.

Furthermore, symptoms of prenatal anxiety also increased the risk of suicidal ideation, which was similar to previous researches [51, 52]. A study also pointed out that more attention should be paid to the anxiety symptoms of pregnant women when preventing suicide [53]. Pregnancy is a state of higher sensitivity for women. They need to deal with the adjustment of the mother's role and the worries after the birth of the fetus, which can lead to some negative thoughts. According to Beck's suicide cognitive model, anxiety interacts with despair to increase attention to suicide [54].

In addition, our results showed that attachment was related to suicidal ideation. The stress model also explained the impact of attachment on perinatal mood disturbance [55]. Psychological factors of pregnant women need to be investigated in the prevention of suicidal ideation. Among those with suicidal ideation, the proportion of insecure attachment was higher and both of attachment anxiety score and attachment avoidance score were higher than those without suicidal ideation group. One explanation can be found in the study of Rohani and collaborators who pointed out that insecure attachment style could increase the susceptibility to suicidal ideation through poor coping style [56].

But the binomial logistic regression analysis only showed the risk factor of suicidal ideation was attachment anxiety. Individuals with high attachment anxiety had a contradictory feeling towards others. Although they yearn for the support of others, they doubt whether they can get the support of others when they need it [57]. Moreover, individuals with high attachment anxiety were more difficult to establish and develop satisfactory relationships [58]. For pregnant women in the third trimester, they may encounter fear of imminent childbirth and physical discomfort associated with pregnancy. But individuals with high attachment anxiety often worry about rejection and abandonment and tend to adopt “over-activation" coping strategies”, thus show a high level of alertness to potential threats and refusal information [59]. The self-representation of attachment anxiety individuals is negative. And those with high attachment anxiety often doubt themselves and express negative emotions such as tension [60]. Pregnant women with high attachment anxiety are more sensitive to the various changes caused by pregnancy and more afraid of loss. Socioeconomic status can be a stress that affects attachment development [61]. Women with attachment anxiety may have more negative emotions because of the increased economic burden after the birth of the fetus, which we will investigate further in the future.

Some potential confounding factors, such as household economic situation, were not examined in this study, especially since the sample of this study is from one of the cities in Anhui Province, which belongs to an economically underdeveloped area. The impact of different economic levels on suicidal ideation needs to be discussed in the future. In addition, Anhui Province is also a patriarchal region, so the impact of neonatal gender expectations on suicidal ideation needs to be considered in the future. Furthermore, the relationship between mother-in-law and daughter-in-law is also a factor worth discussing in the future. Because pregnant women in China have a tradition of confinement after giving birth, they are required not to go out for a month and are usually taken care of by their mother-in-law.

### Limitations

Some limitations must be acknowledged in our research. First, due to the cross-sectional study, the causal relationship between suicidal ideation and related variables cannot be directly inferred. Second, the use of self-report questionnaires may cause some selection bias and sensitive and stigmatizing underreporting. Third, in this study, the assessment of suicide ideation of pregnant women was only based on one item in the EPDS scale, and the systematic suicide scale such as Beck Scale for Suicide Ideation (BSS) was not used for evaluation, which needs to be improved in our future study. For example, further more comprehensive suicide risk assessment on suicidal ideation for pregnant women who are positive for item 10 of the EPDS. Finally, we only discussed the suicidal ideation and related factors of pregnant women in the late pregnancy, the suicidal ideation at different stages of pregnancy the and the influence of prenatal suicidal ideation on postpartum suicidal ideation have not been investigated, which need to be further explored in future studies. Despite these limitations, our study provides some new insights for the prevention of suicidal ideation during pregnancy, especially from the perspective of attachment. Existing research has pointed out that antenatal care could provide some opportunities for the detection and treatment of suicidal ideation.

## Conclusions

Suicidal ideation is common in the third trimester, especially in pregnant women with poor marital satisfaction, high attachment anxiety, prenatal depression and prenatal anxiety. The study of Arachchi et al. also emphasized the need to make suicidal ideation as a priority of emergency intervention. Therefore, in order to reduce prenatal suicidal ideation, regular antenatal examination and appropriate intervention are very necessary. In addition to psychological conditions such as anxiety and depression, the psychological factors such as attachment, also need to be paid attention to in prenatal care. Of course, it is also very important to emphasize the importance of marriage relationship in prenatal mental health care. Individual intervention measures should be formulated for high-risk pregnant women to promote desirable pregnancy outcome.

## Data Availability

All data generated or analyzed during this research period are included in this published article and can be obtained from the corresponding author upon reasonable request.

## Abbreviations

EPDS: Edinburgh Postpartum Depression Scale
SAS: Self-Rating Anxiety Scale
ECR: Experience of Close Relationship
OR: Odds Ratio
CI: Confidence Interval
